# Arsenic Exposure, Arsenic Metabolism, and Glycemia: Results from a Clinical Population in New York City

**DOI:** 10.3390/ijerph18073749

**Published:** 2021-04-03

**Authors:** Fen Wu, Yu Chen, Ana Navas-Acien, Michela L. Garabedian, Jane Coates, Jonathan D. Newman

**Affiliations:** 1 Department of Population Health, New York University School of Medicine, New York, NY 10016, USA; Fen.Wu@nyulangone.org (F.W.); Yu.Chen@nyulangone.org (Y.C.); 2 Department of Environmental Medicine, New York University School of Medicine, New York, NY 10016, USA; 3 Department of Environmental Health Sciences, Columbia University Mailman School of Public Health, New York, NY 10032, USA; an2737@cumc.columbia.edu; 4 Division of Cardiology and the Center for the Prevention of Cardiovascular Disease, Department of Medicine, New York University School of Medicine, New York, NY 10016, USA; Michela.Garabedian@nyulangone.org (M.L.G.); Jane.Coates@nyulangone.org (J.C.)

**Keywords:** urinary arsenic, metabolites, type 2 diabetes, prediabetes, HbA1c, clinics

## Abstract

Little information is available regarding the glycemic effects of inorganic arsenic (iAs) exposure in urban populations. We evaluated the association of total arsenic and the relative proportions of arsenic metabolites in urine with glycemia as measured by glycated blood hemoglobin (HbA1c) among 45 participants with prediabetes (HbA1c ≥ 5.7–6.4%), 65 with diabetes (HbA1c ≥ 6.5%), and 36 controls (HbA1c < 5.7%) recruited from an academic medical center in New York City. Each 10% increase in the proportion of urinary dimethylarsinic acid (DMA%) was associated with an odds ratio (OR) of 0.59 (95% confidence interval (CI): 0.28–1.26) for prediabetes, 0.46 (0.22–0.94) for diabetes, and 0.51 (0.26–0.99) for prediabetes and diabetes combined. Each 10% increase in the proportion of urinary monomethylarsonic acid (MMA%) was associated with a 1.13% (0.39, 1.88) increase in HbA1c. In contrast, each 10% increase in DMA% was associated with a 0.76% (0.24, 1.29) decrease in HbA1c. There was no evidence of an association of total urinary arsenic with prediabetes, diabetes, or HbA1c. These data suggest that a lower arsenic methylation capacity indicated by higher MMA% and lower DMA% in urine is associated with worse glycemic control and diabetes. Prospective, longitudinal studies are needed to evaluate the glycemic effects of low-level iAs exposure in urban populations.

## 1. Introduction

The population of patients with dysglycemia including prediabetes (defined as an HbA1c of 5.7% to 6.4%) and type 2 diabetes (T2D) continues to grow [1]. There remains an unmet clinical need to identify potentially modifiable risk factors for dysglycemia. Exposure to inorganic arsenic (iAs) may be a novel target, as observational and experimental studies have found iAs to have adverse glycemic effects [2,3]. However, there are little to no data on the glycemic effects of iAs exposure in multi-ethnic, urban populations.

Exposure to iAs through drinking water and dietary sources (rice, grains and fruit juice) is common in the US [4,5,6,7,8]. Evidence from early studies in Taiwan and Bangladesh supports an association of high arsenic levels in drinking water (≥150 µg/L) with T2D [9,10,11,12,13], although most studies have been ecological [2]. At low-to-moderate levels of arsenic in drinking water (<50 µg/L), cross-sectional and prospective evidence from the United States, Mexico, and Denmark has shown mixed results regarding the possible role of iAs exposure in T2D [14,15,16,17,18]. Some studies that used total urinary arsenic levels as a biomarker of exposure reported no association between arsenic exposure and T2D or prediabetes [17,19,20], while others reported a positive dose–response relationship [3,18,21,22,23,24,25,26].

The toxicity of iAs is influenced by its metabolism [27]. After absorption, iAs is metabolized into monomethylarsonic acid (MMA) and dimethylarsinic acid (DMA), which are excreted in urine along with unmetabolized iAs. A reduced methylation capacity, characterized by a higher proportion of MMA (MMA%) and lower proportion of DMA (DMA%) in urine, has been identified as a risk factor for skin lesions, cardiovascular disease, and skin, lung, and bladder cancer [28,29,30,31,32,33,34,35]. In contrast, two studies reported that a higher methylation capacity, characterized by lower MMA% and higher DMA% in urine, was related to an increased risk of T2D [18,20]. Two other studies found no association between arsenic methylation capacity and dysglycemia [19,36]. No study has examined the association of urinary markers of iAs metabolism and dysglycemia in a multi-ethnic, urban population in a clinical setting; however, in this population, rates of diabetes and diabetic complications are among the highest in the United States [37]. An improved understanding of potential associations between urinary biomarkers of iAs exposure and dysglycemia in an urban population could further improve our understanding of the pathogenesis of diabetes and possibly uncover novel treatment or control pathways.

In the present study, we investigated the associations of total urinary arsenic concentration and urinary composition of arsenic metabolites with diabetes, prediabetes, and HbA1c in an urban clinical population.

## 2. Methods

### 2.1. Study Population

Patients were recruited as part of a protocol investigating the effects of iAs exposure on glycemic control and non-invasively measured vascular function. Study participants were identified by screening the electronic medical record (EMR) of outpatient visits in the offices of the NYU Faculty Group Practice and the New York Health and Hospital’s Corporation Bellevue Hospital Adult Primary Care Clinic clinics. The EMR was reviewed for patients with normoglycemia, prediabetes (HbA1c 5.7–6.4%), or diabetes (HbA1c ≥ 6.5%) in the 6 months prior to enrollment; patients also met other study inclusion and exclusion criteria seen in outpatient practices. Other recruitment sources include ad-hoc referrals and the public posting of Institutional Review Board-approved flyers in the outpatient clinics. Full inclusion and exclusion criteria are listed in Supplemental Table S1. The study was conducted according to the guidelines of the Declaration of Helsinki, and approved by the Institutional Review Board of New York University School of Medicine (i15-00725; 30 July 2015). Informed consent was obtained from all subjects involved in the study. A total of 2411 patients were identified as eligible through EMR screening and pre-specified as control, pre-diabetic, or diabetic. From the pool of eligible patients, 967 were approached during their clinic appointment or contacted by phone by a member of the study team. Of these 967, reasons for non-participation or ineligibility are listed in Supplemental Table S2. Of the 967 patients contacted for potential enrollment, 423 scheduled a study appointment and data from 190 participants were collected (Supplemental Table S2). Of these, 62 patients had prediabetes, 84 patients had diabetes and 44 were patients without prediabetes or T2D. Study procedures, biospecimen collection and storage were reviewed with study participants who provided signed informed consent. A member of the research team also conducted a face-to-face interview and reviewed the clinical history, demographic information, and medication use of the individual.

### 2.2. Clinical and Demographic Data

HbA1c measurements used to categorize study participants were taken within six months prior to enrollment in the study. Parameters such as race, smoking status, current medications, and history of chronic kidney disease, heart attack, or stroke and other exclusion criteria were obtained from the EMR and confirmed during the interview. Body mass index (BMI), waist circumference, and family history of heart attack or stroke were obtained during the interview.

### 2.3. Arsenic Measurements

Spot urine samples were collected in an arsenic and metal-free cryovial at recruitment and stored at −80 °C. Total urinary arsenic was measured by inductively coupled plasma mass spectrometry (ICPMS) using a Perkin–Elmer NexION 350S ICP-MS (PerkinElmer, Waltham, MA, USA) at the Trace Metals Core Laboratory at Columbia University [38]. Urinary arsenic species (iAs, MMA, DMA, and the combination of arsenobetaine and arsenocholine (AsBC)) were measured on a Perkin–Elmer high performance liquid chromatography (HPLC) Series 200 Pump and Series 200 Autosamplers coupled to a Perkin–Elmer ELAN DRC II ICPMS [38]. Details on the methodology, including analytical standards, reference materials, and quality assurance/quality control, were described previously [39]. The limit of detection (LOD) for all arsenic species was 0.1 µg/L. The percentages of individuals with urinary concentrations below the LOD were 23.8%, 10.2%, 0%, and 3.4% for iAs, MMA, DMA, and AsBC, respectively. These participants were assigned a level equal to the LOD divided by the square root of 2 [40]. Urinary creatinine levels were measured by an automated alkaline picrate reagent method.

Urinary concentrations of total arsenic and arsenic metabolites (iAs, MMA, DMA, and AsBC) were adjusted for urinary creatinine to account for variations in hydration status [41] and expressed as micrograms per gram of creatinine. AsBC is considered to be non-toxic and is present as a large proportion of total arsenic in seafood [40]. Indicators of arsenic metabolism capacity were evaluated as the relative proportions of iAs, MMA, and DMA (iAs%, MMA%, and DMA%) in urine, which were calculated by dividing each metabolite by the sum of iAs, MMA, and DMA [33]. To further remove the influence of AsBC, we also used the residual method to regress the measured total arsenic, iAs%, MMA%, and DMA% on AsBC concentrations and extracted the residuals from the regression model. The residuals reflect total arsenic, iAs%, MMA%, and DMA% not explained by AsBC [42,43].

### 2.4. Statistical Analyses

Of the 190 recruited participants, 146 and 139 participants, respectively, with complete data on total arsenic and species, creatinine, and AsBC were included in the final analysis of diabetes and HbA1c. There were no significant differences in terms of demographic, lifestyle, and diabetes variables between included (*n* = 146) and non-included (*n* = 44) participants (data not shown). We conducted descriptive statistics for socio-demographic and As exposure variables, with the median and interquartile range for continuous variables and distribution (%) for categorical variables, in the overall study population and by diabetes status (control, prediabetes, and diabetes). We used Chi-square and Kruskal–Wallis tests to detect group differences in categorical and continuous variables, respectively.

We used polytomous (generalized logit) logistic regression models to estimate odds ratios (ORs) for prediabetes and diabetes, using participants with a normal HbA1c as the referent group, in relation to a 1 µg/g creatinine increase in urinary AsBC-adjusted total arsenic and a 10% increase in urinary AsBC-adjusted iAs%, MMA%, and DMA%. Since we were interested in understanding the glycemic effects of iAs exposure, we also combined participants *a priori* with abnormal glycemic control (prediabetes and diabetes) into one group and estimated ORs for this group relative to participants with normoglycemia. We first adjusted for sex and age (years) (model 1); then, we additionally adjusted for BMI (weight (kg)/height (m^2^)), smoking status (never vs. ever), and educational attainment (lower than high school vs. greater than high school) (model 2).

We used linear regression models to estimate the difference in HbA1c associated with a 1 µg/g creatinine increase in urinary AsBC-adjusted total arsenic and a 10% increase in urinary AsBC-adjusted iAs%, MMA%, and DMA%, with the adjustment for covariates in model 1 and model 2 above. All analyses were performed using SAS (version 9.4, SAS Institute Inc., Cary, NC, USA).

## 3. Results

Table 1 presents the characteristics of the study population overall and by diabetes status. Overall, approximately 40% of study participants were male and 51% were Hispanic, with a mean age of 56 years and a mean BMI of 30.5 kg/m^2^. Prior smoking was common (40%), and more than half (53%) had an education level greater than high school. Participants with prediabetes were significantly younger than control participants and those with diabetes (Table 1). Control patients had a higher educational level than participants with abnormal glycemic control (prediabetes or diabetes). Urinary AsBC concentrations were lower in participants with prediabetes and diabetes than in controls, reflecting lower seafood intake in participants with dysglycemia relative to controls. There were no significant differences in measures of iAs exposure or metabolites by diabetes status.

There was a positive trend in the association of iAs% with prediabetes and diabetes. Every 10% increase in iAs% was associated with increased odds of prediabetes (OR: 2.89; 95% CI: 0.67–12.5) and diabetes (OR: 3.42; 95% CI: 0.86–13.6; Table 2). Every 10% increase in MMA% was also positively associated with prediabetes (OR: 1.67; 95% CI: 0.54–5.19) and diabetes (2.50; 95% CI: 0.88–7.13). In contrast, each 10% increase in DMA% was associated with lower odds of prediabetes (OR: 0.59; 95% CI: 0.28–1.26). Each 10% increase in DMA% was significantly associated with a ~50% reduction in the odds of diabetes (OR: 0.46; 95% CI: 0.22–0.94) and dysglycemia (prediabetes and diabetes; OR: 0.51; 95% CI: 0.26–0.99). There was no evidence of an association of AsBC-adjusted total arsenic with prediabetes and diabetes.

There was a positive association between urinary MMA% and HbA1c (Table 3). For every 10% increase in MMA%, there was a 1.13% (95% CI: 0.39, 1.88) increase in HbA1c (Model 2). DMA% was inversely associated with HbA1c, and every 10% increase in DMA% was related to a 0.76% (95% CI: 0.24, 1.29) decrease in HbA1c (Model 2).

## 4. Discussion

In this case-control clinical study from a predominantly non-white, Hispanic population receiving medical care in a large, multi-institutional urban medical center, arsenic methylation capacity, but not total urinary arsenic, was associated with dysglycemia. A 10% increase in MMA% was associated a 1.13% increase HbA1c, and a 10% increase in DMA% was associated a 0.76% decrease in HbA1c. We also observed an inverse association of urinary DMA% with prediabetes and diabetes separately and combined. Additionally, we observed a non-significant trend towards increased odds of dysglycemia (prediabetes, diabetes or both) for each 10% increase in MMA%.

Associations between urinary arsenic and diabetes or glycemia in populations with low-to-moderate iAs exposure have been inconsistent. Some cross-sectional studies have indicated that urinary arsenic was positively associated with prevalent diabetes or prediabetes in the US, Canada, and Spain [3,22,25,44,45], while others have shown inconsistent associations between urinary arsenic and HbA1c [3,17,25,44]. In prospective cohort studies among the predominantly rural American Indian population, total urinary arsenic (median levels of 5.9, 21.1, and 10.2 μg/L, respectively) was positively associated with diabetes in some [24,36] but not all analyses [20].

Most prior studies assessing urinary arsenic in relation to diabetes or glycemia were conducted in non-urban populations, with drinking water as the main exposure source and low seafood consumption indicated by AsB (median ranging from 0.3–0.8 µg/L); median urinary arsenic ranged from 5.9–21.1 µg/L in these studies [3,20,24,36]. Compared to these studies, our urban study population showed a similar level of urinary arsenic; the median urinary arsenic levels were 5.0 (IQR: 2.5–7.8) and 17.8 (IQR: 9.6–32.8) µg/L among participants with low seafood consumption (AsBC < 1 µg/L) [40] and those with higher seafood consumption (AsBC ≥ 1 µg/L), respectively. Similar to some previous studies [17,19,20], we did not find an association of total urinary arsenic with diabetes or prediabetes. We acknowledge that our sample sizes were limited in our analyses considering diabetes or prediabetes as a dichotomous outcome (HbA1c ≥ 6.5% or ≥5.7–6.4%) as opposed to smaller effects of arsenic exposure on glycemic control.

Increasing evidence suggests an association between arsenic metabolism, dysglycemia, and diagnosed diabetes, although the nature of this association is not clearly understood [18,20]. Other studies have shown a positive relationship between lower arsenic methylation capacity (increased urinary MMA% and lower DMA%) and health outcomes including cancer and incident cardiovascular disease [30,31,33,35]. In contrast to prior studies [18,20], we observed an association between lower methylation capacity (higher urinary MMA% and lower DMA%) and dysglycemia. Experimental evidence demonstrates that the majority of pancreatic As accumulation is in the form of MMA and DMA [46]. Some studies have shown that 24 h of exposure to subtoxic concentrations of toxic MMA^III^ (1 µM) and DMA^III^ (2 µM) could inhibit insulin-stimulated glucose uptake in cultured adipocytes [47] and decrease glucose-induced insulin secretion in isolated pancreatic islets (0.1 µM MMA^III^/DMA^III^) [48]. Another study demonstrated that 24 h of exposure to MMA^III^ at both 0.375 and 0.5 µM decreased glucose-stimulated insulin secretion in cultured pancreatic β cells [49]. There was, however, no significant decrease observed upon 24 h of DMA^III^ exposure, even at the highest dose (2 µM) [49]. These data indicate the stronger detrimental effects of MMA^III^ relative to DMA^III^ on glucose homeostasis. Using urinary MMA% and DMA% as indicators of methylation capacity and amounts of MMA^III^ and DMA^III^, our findings are consistent with these data. However, in epidemiologic studies, it is not possible to distinguish and quantify MMA^III^, MMA^V^, DMA^III^, and DMA^V^ separately in urine. MMA^III^ and DMA^III^ are very unstable in human urine and must be measured or stabilized immediately after collection, making these impractical for most human studies.

Our study is among the first to report an association of MMA% and DMA% with HbA1c and diabetes in an urban population. Other strengths of this study include the objective assessment of prediabetes and diabetes based on blood HbA1c, the rigorous laboratory procedures and the low limit of detection of our assay for urinary arsenic and its metabolites [39], and standardized study protocols to determine relevant potential confounders. Our results should be interpreted cautiously given the case-control design of the study, which precludes us from determining temporality and thus limits any inferences about causality. We adjusted for several potential confounding factors, but the effect of unmeasured potential confounders such as other environmental/dietary factors or residual confounding remains a possibility. We also had a small number of controls, which may give our results limited statistical power. Urinary arsenic and its metabolites were measured in a single-spot urine sample, which cannot account for physiologic variation in urinary iAs metabolism and excretion. However, under chronic conditions of exposure, urine biomarkers for metals (e.g., As) can also serve as a proxy of long-term exposure [32,50,51]. In addition, HbA1c was measured prior to collection of urine samples and may introduce non-differential measurement errors. However, HbA1c reflects average glycemia over approximately 3 months, and according to the guidelines of the endocrine society, the measurement of HbA1c should occur biannually during stable glycemic control [52], which was applicable to participants in this study. Therefore, we do not expect that HbA1c fluctuated substantially between the measurement of A1c and collection of urine specimens for analysis. To account for urinary dilution, we adjusted for urinary creatinine levels, but this approach may be subject to reverse causality since diabetes is associated with renal impairment, which may affect creatinine excretion [2]. However, participants with abnormal renal function were excluded from our study.

## 5. Conclusions

Our results indicate that lower arsenic methylation, characterized by higher MMA% and lower DMA% in urine, was associated with dysglycemia. Prospective, longitudinal studies are needed to evaluate the impact of arsenic metabolism on glycemic control and diabetes, especially in populations with low levels of iAs exposure.

## Figures and Tables

**Table 1:** Distribution of population characteristics by diabetes status.

Variable *^a^*	N	Overall (*n* = 190)	Controls (*n =* 44)	Prediabetes (*n* = 62)	Diabetes (*n* = 84)	*p*-Value *^b^*
Male	190	72 (37.9)	16 (36.4)	27 (43.6)	29 (34.5)	0.69
Hispanic	190	97 (51.1)	15 (34.1)	44 (71.0)	38 (45.2)	0.64
Age, years	190	56 (51–64)	58 (51.5–64)	52.5 (47–61)	58 (53–64)	0.01
BMI, kg/m^2^	187	30.5 (27.0–34.1)	29.1 (26.0–32.0)	30.4 (26.1–34.5)	31.0 (28.3–34.3)	0.07
Waist circumference, cm	186	103.1 (95.7–111.0)	97.5 (88.5–108.0)	102.4 (97.3–111.0)	106.2 (98.1–111.8)	0.02
Ever smoked	189	75 (39.7)	20 (45.5)	24 (38.7)	31 (37.4)	0.40
Greater than high school	189	100 (52.9)	32 (72.7)	27 (43.6)	41 (49.4)	0.03
Urinary creatinine, mg/dl	178	106.8 (60.4–171.3)	97.5 (46.0–156.1)	132.1 (64.7–187.6)	102.2 (60.7–179.0)	0.19
Hemoglobin A1c, %	182	6.4 (5.8–8.4)	5.5 (5.4–5.5)	6.0 (5.8–6.2)	8.5 (6.9–9.9)	<0.0001
Total arsenic, µg/L	150	13.3 (6.6–27.7)	13.4 (6.4–35.7)	15.5 (8.1–34.0)	12.1 (6.6–23.6)	0.78
iAs, µg/L	146	0.5 (0.3–0.8)	0.5 (0.1–0.7)	0.6 (0.4–0.8)	0.4 (0.3–0.8)	0.24
MMA, µg/L	146	0.7 (0.4–1.0)	0.6 (0.5–0.9)	0.7 (0.4–1.0)	0.8 (0.4–1.2)	0.36
DMA, µg/L	146	6.0 (3.1–10.8)	6.0 (2.8–11.1)	5.8 (3.6–10.7)	6.1 (3.1–9.8)	0.97
AsBC, µg/L	146	4.1 (1.2–11.3)	5.8 (2.0–19.0)	4.1 (1.2–12.0)	3.0 (1.0–8.0)	0.11
iAs + MMA + DMA, µg/L	146	7.4 (3.9–12.8)	7.5 (3.7–13.2)	7.3 (4.4–12.7)	7.4 (3.9–12.3)	0.94
Total arsenic, µg/g creatinine	150	12.0 (6.8–29.2)	15.6 (8.5–40.1)	11.7 (6.8–30.4)	11.3 (6.0–23.1)	0.11
iAs, µg/g creatinine	146	0.4 (0.2–0.7)	0.4 (0.2–0.6)	0.5 (0.3–0.7)	0.4 (0.2–0.7)	0.59
MMA, µg/g creatinine	146	0.6 (0.4–1.0)	0.7 (0.4–1.0)	0.6 (0.4–0.9)	0.6 (0.5–1.0)	0.73
DMA, µg/g creatinine	146	5.2 (3.3–9.0)	6.6 (4.0–12.3)	4.9 (3.3–8.1)	4.5 (3.1–9.0)	0.32
AsBC, µg/g creatinine	146	4.2 (1.2–11.9)	7.5 (3.6–25.4)	3.8 (1.0–9.5)	2.7 (1.1–7.5)	0.01
iAs + MMA + DMA, µg/g creatinine	146	6.7 (4.1–10.7)	7.7 (4.7–13.4)	6.0 (3.9–9.4)	5.5 (4.0–11.1)	0.33
iAs%	146	6.3 (4.0–8.8)	5.7 (3.0–8.6)	6.8 (5.2–9.5)	6.2 (3.9–8.6)	0.17
MMA%	146	10.4 (6.3–13.2)	8.2 (5.9–13.7)	10.4 (7.2–11.8)	10.9 (6.6–13.2)	0.50
DMA%	146	83.7 (78.4–88.1)	86.3 (78.8–90.5)	83.3 (78.4–86.1)	82.1 (78.4–88.1)	0.26

*^a^* Variables are shown as *n* (%) or median (interquartile range (IQR)) as appropriate. *^b^ p* values were computed with the Chi-square or Kruskal–Wallis test to detect group differences in categorical and continuous variables, respectively, among controls and respondents with prediabetes and diabetes. MMA: monomethylarsonic acid; DMA: dimethylarsinic acid; AsBC: the combination of arsenobetaine and arsenocholine.

**Table 2:** Association (odds ratio (OR) *^a^* (95% confidence interval (CI)) between urinary arsenic and metabolism indices and diabetes.

		Prediabetes (*n =* 45) vs. Control (*n =* 36)	Diabetes (*n =* 65) vs. Control (*n =* 36)	Diabetes + Prediabetes (*n =* 110) vs. Control (*n =* 36)
Total arsenic *^b^*				
	Model 1 *^c^*	1.01 (0.97–1.05)	0.98 (0.95–1.02)	0.99 (0.96–1.03)
	Model 2 *^d^*	1.01 (0.96–1.05)	0.99 (0.95–1.03)	0.99 (0.96–1.03)
iAs% *^b^*				
	Model 1 *^c^*	2.05 (0.52–8.08)	2.09 (0.59–7.38)	2.08 (0.65–6.69)
	Model 2 *^d^*	2.89 (0.67–12.5)	3.42 (0.86–13.6)	3.14 (0.87–11.3)
MMA% *^b^*				
	Model 1 *^c^*	1.53 (0.52–4.50)	2.21 (0.85–5.76)	1.96 (0.81–4.79)
	Model 2 *^d^*	1.67 (0.54–5.19)	2.50 (0.88–7.13)	2.19 (0.83–5.76)
DMA% *^b^*				
	Model 1 *^c^*	0.67 (0.33–1.38)	0.56 (0.29–1.08)	0.59 (0.32–1.09)
	Model 2 *^d^*	0.59 (0.28–1.26)	0.46 (0.22–0.94)	0.51 (0.26–0.99)

*^a^* ORs for a 1 µg/g creatinine increase in total arsenic, and a 10% increase in urinary iAs%, MMA%, and DMA%. *^b^* Adjusted for creatinine-adjusted arsenobetaine and arsenocholine using the residual method. *^c^* Adjusted for sex and age (years). *^d^* Adjusted for Model 1 variables plus body mass index, smoking status (never and ever), and educational attainment (lower than high school and greater than high school).

**Table 3:** Association (β *^a^* (95% CI)) between urinary arsenic and metabolism indices and serum hemoglobin A1c.

Variable	*n*	Model 1 *^b^*	Model 2 *^c^*
Total arsenicb *^d^*	139	−0.01 (−0.04, 0.02)	−0.01 (−0.03, 0.02)
iAs% *^d^*	139	0.60 (−0.41, 1.61)	0.75 (−0.28, 1.78)
MMA% *^d^*	139	1.03 (0.31–1.76)	1.13 (0.39–1.88)
DMA% *^d^*	139	−0.67 (−1.19, −0.16)	−0.76 (−1.29, −0.24)

*^a^* β coefficients for a 1 µg/g creatinine increase in total arsenic, and a 10% increase in urinary iAs%, MMA%, and DMA%. *^b^* Adjusted for sex and age (years). *^c^* Adjusted for Model 1 variables plus body mass index, smoking status (never and ever), and educational attainment (lower than high school and greater than high school). *^d^* Adjusted for creatinine-adjusted arsenobetaine and arsenocholine using the residual method.

## Data Availability

The data presented in this study are available on request from the corresponding author. The data are not publicly available due to privacy.

## Supplementary Materials

The following are available online at https://www.mdpi.com/article/10.3390/ijerph18073749/s1, Table S1: Inclusion and exclusion criteria, Table S2: Reasons for lack of participation or ineligibility.
