# Supplementary material for: Arsenic Exposure, Arsenic Metabolism, and Glycemia: Results from a Clinical Population in New York City
Source: Int J Environ Res Public Health. 2021 Apr 3;18(7):3749. doi: 10.3390/ijerph18073749 (PMC8038318; doi:10.3390/ijerph18073749)
Supplement: Supplementary file 1 [file ijerph-18-03749-s001.pdf]

Table S1. Inclusion and exclusion criteria.

**a.**	**Inclusion Criteria**
**1.**	Disordered Glucose Homeostasis
i.	Group A: Prediabetes, A1c 5.7-6.4%
ii.	Group B:
	1. T2D A1c 6.5-7.9% without T2D medications
	2. T2D A1c ≥ 8% with/without T2D medications
**2.**	Control patients (Group C): Normal glucose homeostasis
**3.**	Age ≥ 18
**4.**	No known cardiovascular, cerebrovascular or peripheral arterial disease
**5.**	Able and willing to provide written informed consent for the study
**b.**	**Exclusion Criteria**
**1.**	Unable to speak Spanish or English
**2.**	Active smoking (within the past year)
**3.**	Autoimmune, rheumatologic or inflammatory disease
**4.**	Known active cancer receiving treatment
**5.**	Pregnancy
**6.**	Anemia (hemoglobin < 9 mg/dl)
**7.**	Chronic kidney disease (CrCl < 30ml/min)
**8.**	Known Coronary Artery Disease (CAD; prior stents or CABG)
**9.**	Congestive Heart Failure
**10.**	Known Peripheral Arterial Disease (PAD; lower extremity revascularization surgery OR lower extremity stenting)
**11.**	Known prior stroke or TIA (mini-sroke or temporary/transient stroke)

Table S2. Reasons for lack of participation or ineligibility.

**Pre-screened and eligible**	**2411**
No-show, cancelled clinic appointment, or missed patient at clinic	1024
Other	211
Declined participation via phone	209
**Patient approached in person at clinic appointment**	**967**
Ineligible at time of approach	60
Unable to contact after first approach	419
Declined to participate	249
Other	25
**Patient scheduled study appointment**	**423**
Cancelled, no show for study appointment, screen failure, or incomplete urinalysis	233
**Accrued**	**190**
Subjects with incomplete metals data	44
**Subjects used for analysis**	**146**
